# Molecular Characterization and Functional Analysis of Cytochrome b5 Reductase (CBR) Encoding Genes from the Carotenogenic Yeast *Xanthophyllomyces dendrorhous*

**DOI:** 10.1371/journal.pone.0140424

**Published:** 2015-10-14

**Authors:** María Soledad Gutiérrez, María Cecilia Rojas, Dionisia Sepúlveda, Marcelo Baeza, Víctor Cifuentes, Jennifer Alcaíno

**Affiliations:** 1 Departamento de Ciencias Ecológicas, Facultad de Ciencias, Universidad de Chile, Santiago, Chile; 2 Departamento de Química, Facultad de Ciencias, Universidad de Chile, Santiago, Chile; The University of Wisconsin - Madison, UNITED STATES

## Abstract

The eukaryotic microsomal cytochrome P450 systems consist of a cytochrome P450 enzyme (P450) and a cytochrome P450 redox partner, which generally is a cytochrome P450 reductase (CPR) that supplies electrons from NADPH. However, alternative electron donors may exist such as cytochrome b5 reductase and cytochrome b5 (CBR and CYB5, respectively) via, which is NADH-dependent and are also anchored to the endoplasmic reticulum. In the carotenogenic yeast *Xanthophyllomyces dendrorhous*, three P450-encoding genes have been described: *crtS* is involved in carotenogenesis and the *CYP51* and *CYP61* genes are both implicated in ergosterol biosynthesis. This yeast has a single CPR (encoded by the *crtR* gene), and a *crtR* ^-^ mutant does not produce astaxanthin. Considering that this mutant is viable, the existence of alternative cytochrome P450 electron donors like CBR and CYB5 could operate in this yeast. The aim of this work was to characterize the *X*. *dendrorhous* CBR encoding gene and to study its involvement in P450 reactions in ergosterol and carotenoid biosynthesis. Two CBRs genes were identified (*CBR*.1 and *CBR*.2), and deletion mutants were constructed. The two mutants and the wild-type strain showed similar sterol production, with ergosterol being the main sterol produced. The *crtR* ^-^ mutant strain produced a lower proportion of ergosterol than did the parental strain. These results indicate that even though one of the two *CBR* genes could be involved in ergosterol biosynthesis, *crtR* complements their absence in the *cbr* ^-^ mutant strains, at least for ergosterol production. The higher NADH-dependent cytochrome c reductase activity together with the higher transcript levels of *CBR*.*1* and *CYB5* in the *crtR* ^-^ mutant as well as the lower NADH-dependent activity in CBS-*cbr*.1^-^ strongly suggest that CBR.1-CYB5 via participates as an alternative electron donor pathway for P450 enzymes involved in ergosterol biosynthesis in *X*. *dendrorhous*.

## Introduction

The cytochrome P450s (P450s) constitute a large superfamily of heme-containing monooxygenases present in organisms from all domains of life [1,2]. They play significant roles in the oxidative metabolism of a wide range of exogenous and endogenous substrates [3]. These enzymes are involved in the metabolism of physiologically important compounds such as sterols, fatty acids and vitamins [4], secondary metabolites [5] and in the activation and detoxification of many xenobiotics such as drugs, carcinogens and environmental pollutants [4,6]. The P450s act as a terminal electron acceptor in the multicomponent P450 dependent monooxygenation system (P450 systems) which leads to the reductive activation of molecular oxygen followed by the insertion of one oxygen atom into the substrate molecule, which catalyzes the following general reaction: RH + O_2_ + 2e^−^ + 2H^+^ → ROH + H_2_O, where R represents the substrate molecule [7]; the required electrons are generally supplied by NADPH and transferred to P450 by a P450 redox partner [8]. In the class II eukaryotic microsomal P450 systems, the general P450 redox partner is a cytochrome P450 reductase, termed CPR [1,7,9], and the electron flow goes from NADPH to FAD to FMN and finally to the heme group in P450. However, alternative electron transfer mechanisms from NADH via cytochrome b5 reductase and cytochrome b5, which are anchored to the endoplasmic reticulum (CBR and CYB5), have been reported [10]. In this last case, the electron flow goes from NADH to CBR to CYB5 and finally to P450, stimulating and increasing P450 reactions [2,11]. Particularly in the fungus *Phanerochaete chrysosporium*, CBR and CYB5 are involved in the function of the P450 CYP63A2 that is capable of oxidizing polycyclic aromatic hydrocarbons, alkyl phenols and alkanes [12]. In *Saccharomyces cerevisiae* it was also demonstrated that CBR and CYB5 could maintain CYP51 activity [13]. In *cpr* ^*-*^ mutant strains of the ascomycete *Fusarium fujikuroi*, the P450s activities involved in gibberellin biosynthesis showed changes in their regioselectivity, reaction rate and formation of alternative products due to their interaction with alternative redox partners [14].

The basidiomycete yeast *Xanthophyllomyces dendrorhous* has an enzyme related to the 3A sub-family member cytochrome P450, astaxanthin synthase CrtS (encoded by the *crtS* gene), which converts beta-carotene into astaxanthin [15,16]. This carotenoid has huge biotechnological potential, as it is currently use in the pharmaceutical and cosmetic industries due to its antioxidant properties and in aquiculture for salmonid fish pigmentation [17]. Astaxanthin synthase has not been reported in other astaxanthin-producing organisms, suggesting that in *X*. *dendrorhous* yeast, a unique P450 system has evolved that is specialized in the synthesis of astaxanthin [16].

In addition to astaxanthin synthase, other two P450 encoding genes have been described in *X*. *dendrorhous* that are involved in ergosterol biosynthesis: *CYP61* [18] and *CYP51* [19]. Sterols are essential structural and regulatory components of eukaryotic cell membranes that modulate their thickness, fluidity and permeability [20]; ergosterol is the principal sterol in yeasts that fulfills similar functions as cholesterol in mammalian cells. Although in most organisms there are several P450 encoding genes, in most species, there is only one CPR encoding gene, with few exceptions [21]. The *X*. *dendrorhous* CPR gene (named as *crtR* in this yeast) was characterized and was shown to be essential for the synthesis of astaxanthin [22]. It has been proposed that the *X*. *dendrorhous* astaxanthin producing cytochrome P450 system (CrtS and CrtR) is also unique, because CrtS has a high specificity for its own CPR, CrtR. The astaxanthin production in metabolically engineered *Saccharomyces cerevisiae* strains was only achieved when *crtS* was co-expressed with *crtR* [23]. Furthermore, according to protein modeling and molecular dynamics simulations, it was predicted that CrtS interacts preferentially with the FMN-binding domain of CrtR rather than with the one of the CPR of *S*. *cerevisiae*, due to a larger interfacial area of interaction and a higher number of hydrogen bonds and saline bridges formed at the interaction surface [24].

Considering the relevance of sterols for the cell, the fact that *crtR* gene disruption is not lethal in *X*. *dendrorhous* [22], indicates the existence of alternative cytochrome P450 electron donors. The aim of this study was to identify and characterize an alternative to the CPR P450 electron donor, specifically the CBR-CYB5 pathway in *X*. *dendrorhous*. Considering that unlike CPR, CBR is preferentially NADH-dependent, *cbr* ^-^ mutants were constructed and analyzed. The obtained results provide evidence that the CBR-CYB5 pathway partially complements the *crtR* ^-^ mutation by fulfilling an alternative P450 redox-partner role.

## Materials and Methods

### Strains and culture conditions

All strains used and constructed in this work are listed in Table 1. The *X*. *dendrorhous crtR* ^-^ mutant strain, CBSTr [22] that derives from the wild-type strain CBS 6938 (ATCC 96594), were used in this work for phenotypic analyses and genetic modifications. The original *X*. *dendrorhous* genomic and transcriptomic sequences were obtained from strain UCD 67–385 (ATCC 24230) by two Next Generation Sequencing (NGS) platforms [25]. Additionally, the genome from strain CBS 6938 was released this year [26].

**Table 1:** Strains and plasmids used and/or constructed in this work.

	Genotype or Relevant Feature	Reference or Source
***Strains*:**		
*E*. *coli*		
DH-5alpha	F- φ80d lacZΔM15Δ (lacZY-argF) U169 deoR recA1 endA1 hsdR17(rk- mk+) phoA supE44l- thi-1 gyrA96 relA1.	[35]
*X*. *dendrorhous*		
UCD 67–385	ATCC 24230, wild-type. Diploid strain [55].	ATCC
CBS 6938	ATCC 96594, wild type.	ATCC
CBSTr	*crtR* ^*-*^ mutant strain from CBS 6938 parental wild-type strain. Hygromycin B resistant mutant.	[22]
CBS*-cbr*.1^-^	*cbr*.1^-^ mutant strain from CBS 6938 parental wild-type strain. Hygromycin B resistant mutant.	This work
CBS*-cbr*.2^*-*^	*cbr*.2^-^ mutant strain from CBS 6938 parental wild-type strain. Zeocin resistant mutant.	This work
***Plasmids*:**		
pBluescript SK- (pBS)	ColE1 ori; AmpR; cloning vector with blue-white selection.	Stratagene
pMN-*hph*	pBS contained at the *Eco*RV site a cassette of 1.8 kb bearing the *E*. *coli*-Hygromycin B resistance (*hph*) gene under EF-1 α promoter and the GPD transcription terminator of *X*. *dendrorhous*.	[56]
pIR-*zeo*	pBS contained at the *Eco*RV site a cassette of 1.2 kb bearing the *Streptoalloteichus hindustanus* Zeocin resistance *Sh ble* gene under the EF-1 α promoter and GPD transcription terminator of *X*. *dendrorhous*.	[18]
pXd-g*CBR*.1::*hph*	pBS contained at the *Eco*RV, 865 pb upstream, 750 pb downstream of the *CBR*.1 gene and the hygromycin B resistance cassette between them.	This work
pXd-g*CBR*.2::*zeo*	pBS contained at the *Eco*RV, 677 pb upstream, 803 pb downstream of the *CBR*.2 gene and the zeocin resistance cassette between them.	This work

ATCC: American Type Culture Collection.

For most experiments, strains CBS 6938, CBSTr, CBS-*cbr*.1::*hph* and CBS-*cbr*.2::*zeo* were cultured independently in three replicates, which were incubated at 22°C with constant agitation in 1 L Erlenmeyer flasks containing 400 mL of YM medium (1% glucose, 0.3% yeast extract, 0.3% malt extract and 0.5% peptone). The growth curves of each strain were constructed by registering the optical density of the cultures at 600 nm with a V-630 UV-Vis Spectrophotometer from JASCO. For the analyses, 65 mL samples were taken for: i) yeast dry weigh determination (2 samples of 5 mL each), ii) carotenoid extraction (one sample of 15 mL), iii) sterol extraction (one sample of 5 mL), iv) obtaining the microsomal fraction (2 samples of 10 mL) for cytochrome c reductase activity assays and v) RNA extraction (4 samples of 5 mL each). The obtained cellular pellets were washed with sterile distilled water and kept at -80°C until the samples were processed.

### Carotenoid and Sterol extraction, and RP-HPLC analyses

Carotenoids and sterols were extracted according to [27] and [28], respectively, quantified spectrophotometrically and normalized to the dry weight of the yeast. Carotenoids were quantified at 465 nm using an absorption coefficient of A_1%_ = 2,100 and sterols at 280 nm were quantified using an absorption coefficient of A_1%_ = 11,500. The extracted carotenoids and sterols were separated by RP-HPLC using an RP-18 Lichrocart 125–4 (Merck) column with acetonitrile: methanol: isopropanol (85:10:5, v/v) and methanol: water (97:3, v/v) as the mobile phase, with a 1 mL/min flux under isocratic conditions. The elution spectra were recovered using a diode array detector; carotenoids and sterols were identified according to their spectra and retention time compared to standards.

### Obtaining the microsomal fraction

The microsomal fraction was obtained from cell pellets from 10 mL of culture suspended in 4 mL of extraction buffer (1 mM EDTA, 50 mM Tris HCl pH 7.5, 1 mM DTT, 0.3 M Sorbitol, Complete^TM^ protease inhibitor cocktail from Boehringer Mannheim). Cells were lysed mechanically by sonication using a Cole Parmer 4710 ultrasonic homogenizer with 500 μL of 0.5 mm glass beads (BioSpec). Twenty sonication pulses of 20 s at 4°C, followed by a 1 min incubation on ice. The cell lysate was centrifuged at 1,000 x g for 3 min and the supernatant was recovered and centrifuged at 20,000 x g at 4°C for 30 min; the new supernatant was recovered and centrifuged at 100,000 x g at 4°C for 1 h. The pellet obtained was suspended in wash buffer (1 mM EDTA, 50 mM Tris HCl pH 7.5, 1 mM DTT, and 0.3 M sorbitol) and centrifuged at 100,000 x g for 1 h at 4°C. Finally, the obtained pellet, which corresponded to the microsomal fraction, was suspended in 200 μL of buffer (30 mM potassium phosphate pH 7.8 and 0.1 mM EDTA) and fractioned into 40 μL samples in Eppendorf tubes, which were frozen with liquid nitrogen and stored at -80°C until use. The protein concentration was determined using the Coomassie Plus Assay Kit (Thermo Scientific) according to the supplier.

### Cytochrome c reductase activity assay

The methodology used was adapted from [29], which measured the cytochrome c absorbance increment at 550 nm when it was reduced. The assay was performed in a 275 μL cuvette (UV-Micro, BRAND) containing 237.5 μL assay solution (260 mM potassium phosphate pH 7.8, 0.09 mM EDTA, BSA 0.01 mg/mL and 31 mM cytochrome c) and 5 to 10 μL of the microsomal fraction sample. The reaction was started by adding 12.5 μL of NADPH (2 mM) or NADH (2 mM) and the absorbance at 550 nm was registered every 1 s during 5 min using the kinetic mode of the V-630 UV-Vis Spectrophotometer from JASCO at 22 ± 1°C. The initial velocities of each progress curve were determined with the JASCO Spectra Analysis software.

### Identification and characterization of the putative CBR encoding gene from *X*. *dendrorhous*

The *CBR* genomic and cDNA sequences from *X*. *dendrorhous* were identified by BLAST analyses over the collection of genomic and transcriptomic contigs and scaffolds of strain UCD 67–385 using the homologous genes from *Saccharomyces cerevisiae*, *Phanerochaete chrysosporium* and *Cryptococcus neoformans* as queries [GenBank: Z28365.1, AY835609.1 and XM_569177.1, respectively]. The nucleotide and deduced amino acid sequences were analyzed with the CLC Genomic Workbench, Geneious 6.0.4, and programs available on-line such as: InterProScan (http://www.ebi.ac.uk/Tools/pfa/iprscan5/ [30]), Tmpred (http://www.ch.embnet.org/software/TMPRED_form.html [31]) and JPRED 3 (http://www.compbio.dundee.ac.uk/www-jpred/ [32]). Phylogenetic analyses were carried out using MEGA v.6.06 [33] with the maximum-parsimony method and 1,000 bootstrap replicates.

### DNA extraction, amplification and sequencing

Total DNA from *X*. *dendrorhous* was extracted according to [34]. All oligonucleotides used in this study were listed in S1 Table and were purchased at Integrated DNA Technologies (IDT). PCR reactions were performed in a 2720 (Applied Biosystems) thermal cycler at a final volume of 25 μL containing 2 U of *Pfu* DNA polymerase (Thermo Scientific), 2.5 μL of 10X *Pfu* buffer, 0.5 μL of 10 mM dNTPs, 1 μL of 50 mM MgCl_2_, 1 μl of each primer (25 μM) and 10 to 20 ng of template DNA. In general, the amplification protocol was: initial denaturation at 95°C for 3 min; 35 cycles of denaturation at 94°C for 30 s, annealing at 55°C for 30 s, and synthesis at 72°C for 3 min; and a final extension step at 72°C for 10 min. Samples were kept at 4°C until analysis by 0.8% agarose gel electrophoresis in TAE buffer containing 0.5 μg/mL ethidium bromide [35]. DNA for sequencing was purified from gels by the glass milk method [36]. Nucleotide sequences were obtained from an ABI 3100 Avant genetic analyzer using the BigDye terminator v3.1 kit (Applied Biosystems).

### RNA extraction, single strand DNA synthesis and quantitative PCR (RT-qPCR)

Total RNA was extracted according to a modified protocol from Chomczynski and Sacchi [37,38] and RNA was quantified spectrophotometrically at 260 nm [35]. The synthesis of cDNA was performed according to the M-MLV reverse transcriptase (Invitrogen) manufacturer’s protocol, with 5 μg of total RNA at a final volume of 20 μL using oligo-dT_18_. The relative transcript level determination was performed in an Mx3000P quantitative PCR system (Stratagene) using primers pairs (S1 Table) with efficiencies greater than 95%, as determined by standard curves with a correlation coefficient of R^2^ ≥ 0.996. Each reaction contained 1 μL of the reverse transcription reaction, 0.25 μM of each primer and 10 μL of the SensiMix SYBR Green I (Quantace) kit at a final volume of 20 μL. The obtained Ct values were normalized to the respective value of beta-actin [GenBank: X89898.1] [39] and were later expressed as a function of the control conditions (wild type strain) using the ΔΔCt algorithm [40].

### Plasmid construction and *X*. *dendrorhous* transformation

All plasmids used in this work were listed in Table 1. To knockout the *X*. *dendrorhous CBR* genes, the plasmids pXd-g*CBR*.1::*hph* and pXd-g*CBR*.2::*zeo* were constructed. Initially, the upstream and downstream DNA region of approximately 850 pb for each gene were PCR-amplified from genomic DNA from strain UCD 67–385 using the primers pairs pre_gCBR1-Fw + gCBR1-HpaI-Rv, gCBR1-HpaI-Fw + post_gCBR1-Rv, cytb5Red_del-Fw + cytb5Red_del_HpaI-Rv, and cytb5Red_del_HpaI-Fw + cytb5Red_del-Rv to amplify the *CBR*.1 upstream, the *CBR*.1 downstream, the *CBR*.2 upstream and the *CBR*.2 downstream regions, respectively. Then, the upstream and downstream DNA regions of each gene were joined by OE-PCR [41] leaving an *Hpa*I restriction site between them according to the design of the primers that were used. The new DNA fragments were ligated into the *Eco*RV site of the plasmid pBluescript SK- [35]. Finally, the plasmids pXd-g*CBR*.1::*hph* and pXd-g*CBR*.2::*zeo* were generated by digesting the previous plasmids with *Hpa*I and introducing an antibiotic resistance cassette (hygromycin B: *hph* or zeocin: *zeo*) for *X*. *dendrorhous* transformant selection.

Electrocompetent *X*. *dendrorhous* cells obtained from exponential cultures (OD_600nm_ = 1.2) grown in YM medium were transformed by electroporation using a Bio-Rad gene pulser X cell with PC and CE modules under the following conditions: 125 mF, 600 Ω, and 0.45 kV. Transformations were performed using 1 to 5 μg of linear donor DNA, which was released from the plasmids pXd-g*CBR*.1::*hph* and pXd-g*CBR*.2::*zeo* by digesting with *Not*I + *Cla*I and *Not*I + *Xho*I, respectively. Yeast transformants were selected on YM-agar plates (1.5%) supplemented with 35 μg/mL hygromycin-B or 40 μg/mL zeocin when necessary.

## Results and Discussion

### Phenotypic analysis of the wild-type and *crtR* ^*-*^ mutant *X*. *dendrorhous* strains

In a previous work, the gene that encodes the *X*. *dendrorhous* CPR (CrtR, *crtR* gene) was identified and was demonstrated to be essential in the production of astaxanthin in this yeast [22]. However, its participation in other pathways involving P450 enzymes, such as ergosterol biosynthesis, was not analyzed. As such, we performed phenotypic analyses focusing on the production of astaxanthin (as control) and ergosterol in the wild-type (CBS 6938) and *crtR* ^-^ mutant strain (CBSTr) derived from CBS 6938. Both strains were cultivated in triplicate and samples were taken along the growth curve (after 21, 38, 72 and 120 h of culture, corresponding to early exponential, middle exponential, early stationary and late stationary phases of growth, respectively) to evaluate the production of carotenoids and sterols. Statistical analyses (Student’s t test, p<0.05) confirmed that the CBSTr strain reached a higher carotenoid content than the wild-type strain after 120 h of culture. However, as was previously reported, CBSTr does not produce astaxanthin but accumulates beta-carotene, indicating that only CrtR supports the electron flow to astaxanthin synthase (CrtS), which belongs to the P450 protein family.

**Fig 1:**
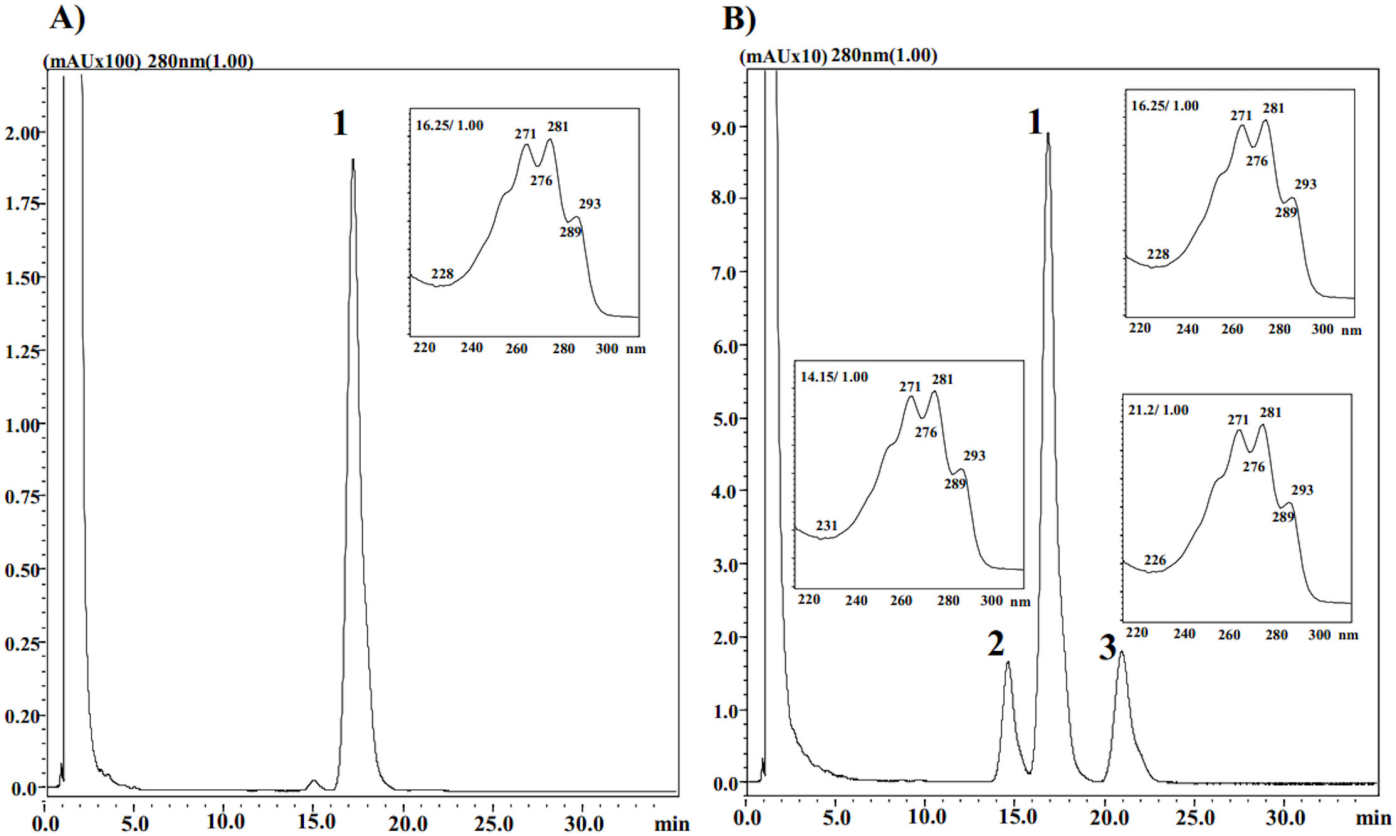
RP-HPLC analysis of sterols from the wild-type and *crtR*- mutant strain. Chromatograms (at 280 nm) correspond to sterols extracted after 72 h of culture from the (A) wild-type (CBS 6938) and (B) CBSTr strain (*crtR* ^-^). The corresponding spectra were displayed beside each peak (peaks N° 1 to 3). Peak 1 corresponds to ergosterol, which was confirmed by co-injecting the sample with standard ergosterol.

In contrast, total sterol production was similar between both strains at all analyzed culture times, but the sterol composition was different. All the sterol samples from the wild-type strain showed a single predominant RP-HPLC peak at the 280 nm channel, with a retention time of approximately 16 min (peak 1) with the ergosterol characteristic UV-spectra (Fig 1A). The identity of this sterol was confirmed by co-injecting each sample with standard ergosterol. On the other hand, three peaks with sterol characteristic UV-spectra at retention times of approximately 15 (peak 2), 16 (peak 1) and 22 (peak 3) min were observed in the RP-HPLC profile of sterol samples from the *crtR* ^-^ mutant strain, with ergosterol (peak 1, also confirmed by co-injecting the samples with standard ergosterol) being the most abundant (Fig 1B). These results show that in *X*. *dendrorhous*, there must be an alternative to the CPR P450 electron donor to sustain the synthesis of ergosterol in CBSTr, although other sterols beside ergosterol are accumulated. This was not the case in *S*. *cerevisiae*, as the total sterol content in a cytochrome P450 reductase mutant strain was approximately 4-fold lower than in the wild-type strain, but the ergosterol fraction in both strains was the same (approximately 90%) [42]. Interestingly, the retention times of peaks 2 and 3 coincided with the two single peaks observed in the sterol samples from the *X*. *dendrorhous cyp61* ^-^ mutant strains [18]. To confirm this observation, sterol samples were obtained from the *crtR* ^-^ and *cyp61* ^-^ mutant strains which were independently co-injected with standard ergosterol and also mixed together, revealing that CBSTr indeed accumulates the same sterols as the *cyp61* ^-^ mutant, besides ergosterol. This result suggests that the functionality of the CYP61 enzyme could be partially affected in the CBSTr mutant strain. However, this strain still produces ergosterol, so there must be a P450 electron donor via an alternative to cytochrome P450 reductase in *X*. *dendrorhous* that complements ergosterol, but not astaxanthin biosynthesis. The results indicated that the alternative electron donor, probably CBR-CYB5, had a different affinity for the P450s enzymes CrtS, CYP51 and CYP61. This agrees with *in vitro* enzymatic assays described in *S*. *cerevisiae* CBR, CYB5 and CYP51, in which it was determined that CYP51 had efficient activity with CBR-CYB5 [13].

### Identification and molecular characterization of the *X*. *dendrorhous CBR* and *CYB5* genes

By BLAST analyses over the genomic and transcriptomic sequences of the UCD 67–385 strain, we were able to identify the putative *X*. *dendrorhous CBR* and *CYB5* genes. Two *CBR* and a single *CYB5* genes were identified, which were named *CBR*.1, *CBR*.2 and *CYB5*, respectively, and were uploaded to the GenBank database [KT448554, KT448556 and KT448555, respectively]. From the sequence analyses of the gDNA and cDNA versions of each gene, their genetic structures were determined.

**Fig 2:**
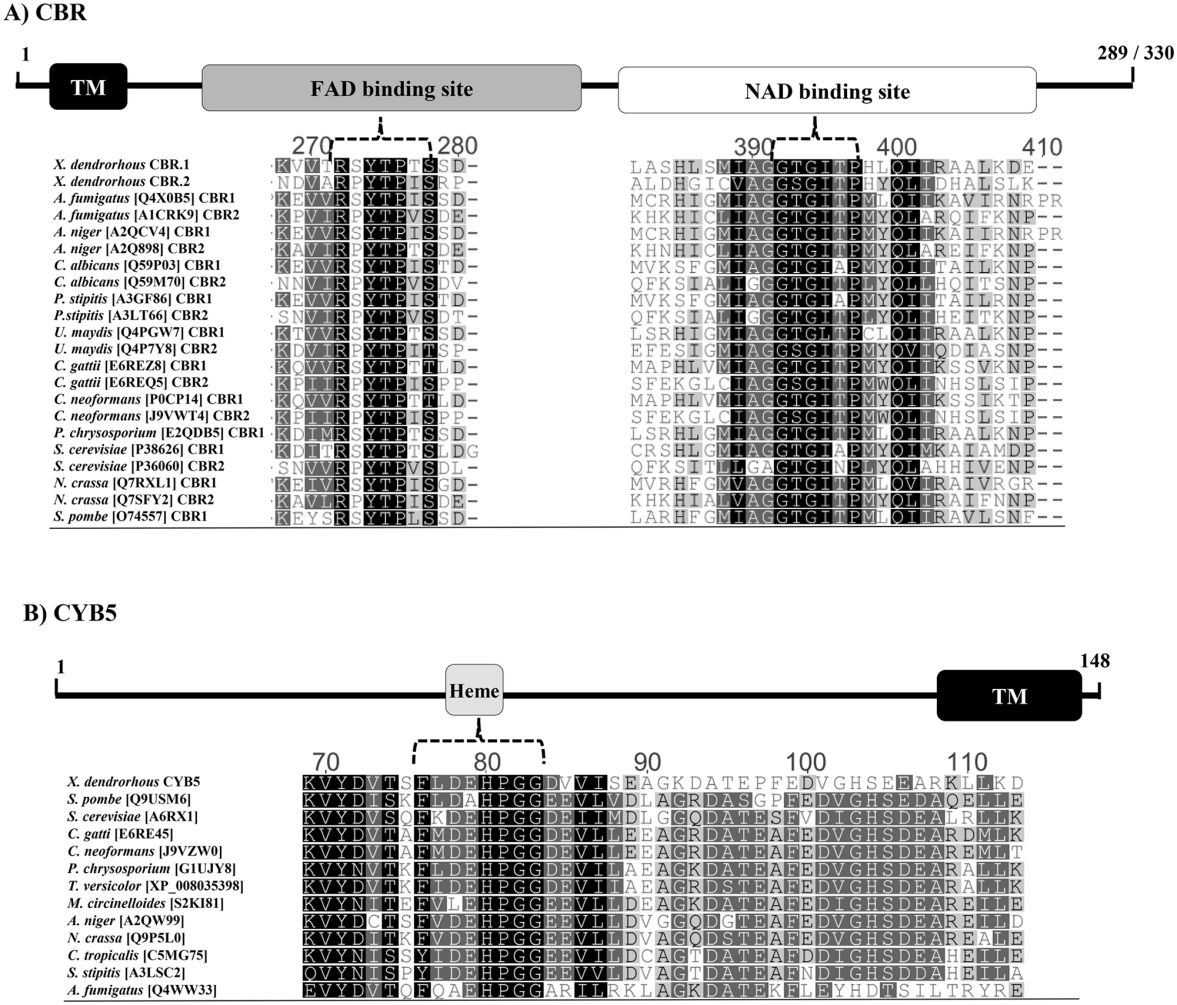
*X*. *dendrorhous* CBR.1, CBR.2 and CYB5 protein amino acid sequence alignment. Amino acid sequence alignment of conserved protein motives: (A) CBR and (B) CYB5. The *X*. *dendrorhous* CBR.1, CBR.2 and CYB5 are included. The UniProtKB or GenBank accession number is indicated in brackets beside the organism name and above each alignment; a scheme of the deduced *X*. *dendrorhous* protein structure is included. Tm: trans-membrane segment.

**Fig 3:**
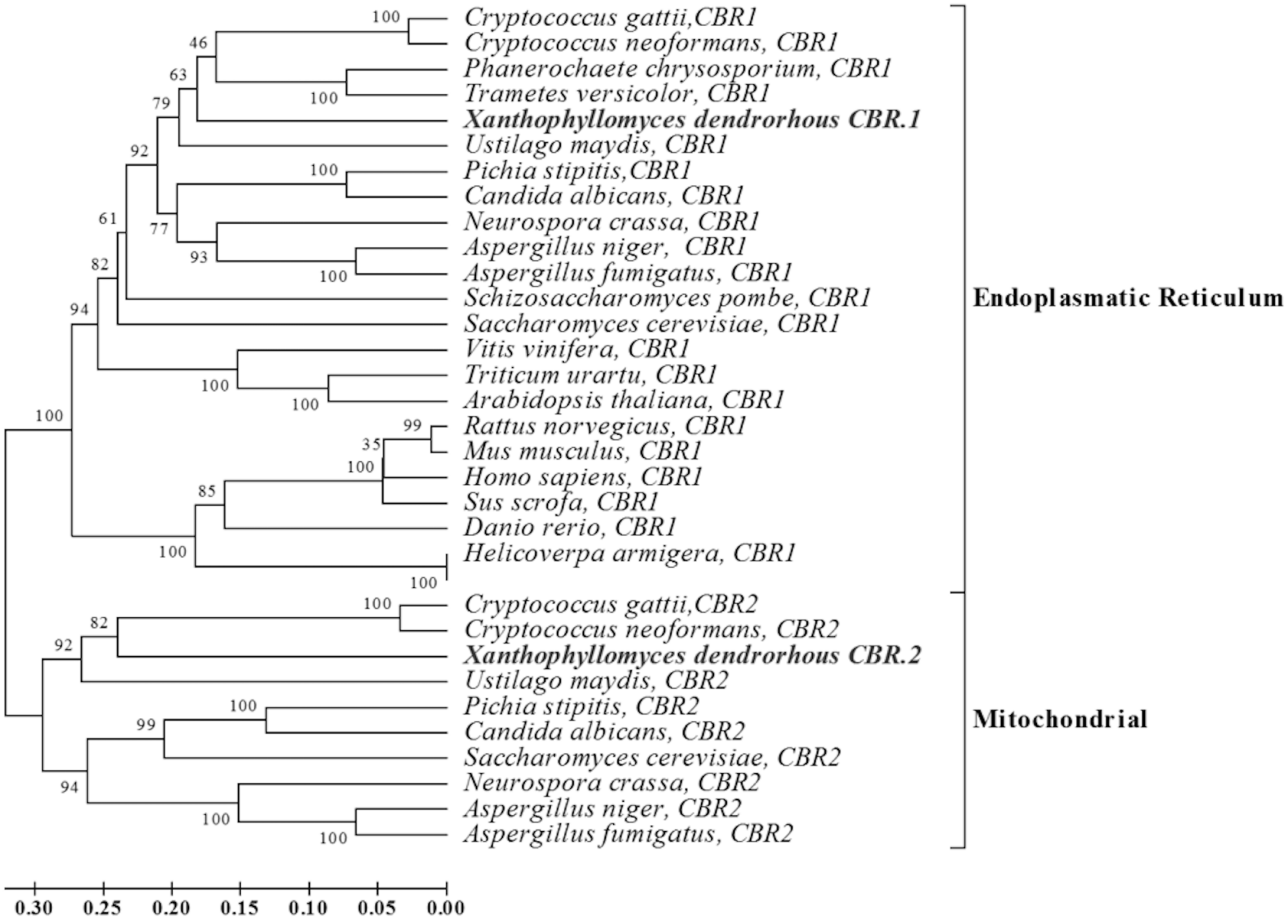
Phylogenetic analysis of the CBR genes from *X*. *dendrorhous*. The accession numbers for each of the amino acid sequences are: *Cryptococcus gattii* CBR1 [E6REZ8] and CBR2 [E6REQ5], *C*. *neoformans* CBR1 [P0CP14] and CBR2 [J9VWT4], *P*. *chrysosporium* CBR1 [E2QDB5], *T*. *versicolor* CBR1 [XP_008033072], *U*. *maydis* CBR1 [Q4PGW7] and CBR2 [Q4P7Y8], *P*. *stipitis* CBR1 [A3GF86] and CBR2 [A3LT66], *C*. *albicans* CBR1 [Q59P03] and CBR2 [Q59M70], *N*. *crassa* CBR1 [Q7RXL1] and CBR2 [Q7SFY2], *A*. *niger* CBR1 [A2QCV4] and CBR2 [A2Q898], *A*. *fumigatus* CBR1 [Q4X0B5] and CBR2 [A1CRK9], *S*. *pombe* CBR1 [O74557], *S*. *cerevisiae* CBR1 [P38626] and CBR2 [P36060], *V*. *vinifera* CBR1 [F6HIY1], *T*. *urartu* CBR1 [M7YU08], *A*. *thaliana* CBR1 [Q9ZNT1], *H*. *armigera* CBR1 [F1CZW3], *D*. *rerio* CBR1 [Q7ZVF8], *S*. *scrofa* CBR1 [F1S4N2], *H*. *sapiens* CBR1 [Q9UHQ9], *M*. *musculus* CBR1 [Q9DB73], and *R*. *norvegicus* CBR1 [G3V9S0]. The UniProtKB or GenBank accession number is indicated in brackets. The value at each node indicates the Bootstrap after 1000 iterations using MEGA v.6.06.

The *X*. *dendrorhous CBR*.1 gene has an ORF of 870 bp and consists of 7 exons of 103, 85, 316, 82, 35, 173, 76, bp and 6 introns of 123, 68, 89, 93, 97 and 132 bp. This gene encodes a predicted CBR protein of 289 amino acids with a molecular weight of 31.48 kDa (pI, 6.96). In contrast, the *CBR*.2 gene has 9 exons of 82, 12, 121, 307, 111, 154, 92, 40 and 74 bp, and 8 introns of 247, 126, 104, 92, 95, 86, 121 and 87 bp, giving an ORF of 993 bp. The *X*. *dendrorhous CBR*.2 gene encodes a predicted 330 amino acid protein with molecular weight of 35.35 kDa (pI, 8.41). Bioinformatic analyses of both deduced *X*. *dendrorhous* CBR proteins also revealed structurally conserved elements in the cytochrome b5 reductase protein family: i) an NADH binding domain (signature: G-x-G-x-x-P), ii) an FAD binding domain (signature: R-x-Y-T-x-x-S) and iii) a transmembrane segment at the amino terminal region, through which CBRs anchors to the endoplasmic reticulum (Fig 2A). Interestingly, the subcellular distribution of both *X*. *dendrorhous* CBR proteins might be different according to the PSORT tool based on the method of McGeoch's [43], which predicted that CBR.1 was likely located in the endoplasmic reticulum, while CBR-2 was located in the mitochondrial membrane. Moreover, by a phylogenetic analysis, it was found that the CBR.1 groups together with other CBRs were most of them have been described as microsomal, while CBR.2 showed a closer relation with CBRs described as mitochondrial (Fig 3). For these reasons, it was most likely that CBR.1, and not CBR.2, would be involved in the class II P450 systems in *X*. *dendrorhous*.

Similarly, the *CYB5* gene has an ORF of 444 bp with 6 exons of 145, 39, 27, 95, 68 and 73 bp, and 5 introns of 227, 105, 115, 87 and 110 bp. This gene encodes a 148 amino acid polypeptide with a predicted molecular weight of 16.07 kDa (pI, 4.87). The deduced protein size was consistent with CYB5 proteins described in other fungi, given that the reported size of this protein ranged from 120 to 158 amino acids [44–46]. Moreover, structural conserved elements in the cytochrome b5 protein family were identified in the deduced amino acid structure of the *X*. *dendrorhous CYB5* gene including: i) a putative hydrophobic transmembrane segment at the carboxyl terminus, which could anchor the protein to the endoplasmic reticulum and ii) the cytochrome b5 family heme-binding domain with the conserved signature: [FY]-[LIVMK]-(I)-(Q)-H-P-[GA]-G [47] (Fig 2B).

### *CBR*.1 and *CBR*.2 gene mutations in *X*. *dendrorhous*

**Fig 4:**
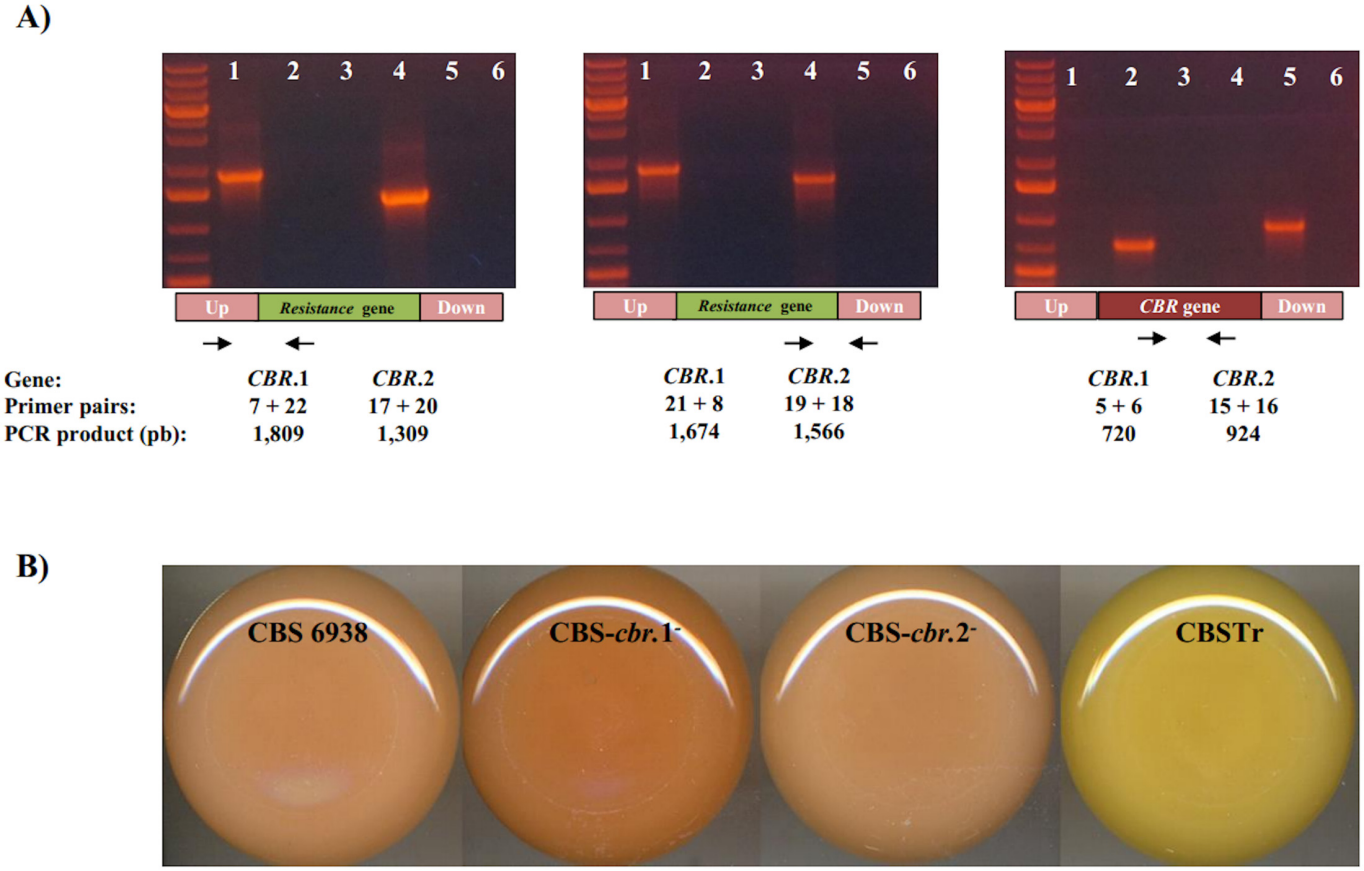
PCR-based analysis of the CBS-*cbr*.1^-^ and CBS-*cbr*.2^-^ strains. (A) Each gel shows the PCR reaction products obtained with different primers pairs numbered according to S1 Table and, as template, genomic DNA from the following strains: CBS-*cbr*.1^*-*^ (line 1), CBS 6938 (line 2), negative primer control (line 3), CBS-*cbr*.2^*-*^ (line 4), CBS 6938 (line 5), and negative primer control (line 6). Under each gel, a general scheme of the PCR analyses is included representing the resistance cassettes (green), the corresponding *CBR* gene (red) and the *CBR* flanking DNA (pink). A 1 kb plus DNA ladder (20.0, 10.0, 7.0, 5.0, 4.0, 3.0, 2.0, 1.5, 1.0, 0.7, and 0.5 Kbp) was used as the molecular weight standard in the right lane of each gel. (B) Color phenotype of the strains CBS 6938 (WT), CBS-*cbr*.1^*-*^, CBS-*cbr*.2^*-*^ and CBSTr after 5 days of culture on YM medium incubated at 22°C with constant agitation.

As CBR is the enzyme responsible for the capture of electrons directly from NADH, it was expected that *CBR*.1 or *CBR*.2 gene mutations would decrease the CBR-CYB5 electron supply by using NADH as a cofactor. The mutant strains CBS-*cbr*.1::*hph* and CBS-*cbr*.2::*zeo* were obtained from the wild-type strain CBS 6938, where the corresponding gene was replaced with an antibiotic resistance marker through a double homologous recombination event (Table 1). According to previous results [22], the wild-type CBS 6938 strain was likely aneuploid, and the CBS-*cbr*.1::*hph* and CBS-*cbr*.2::*zeo* strains were hemizygous as PCR-based genotype analysis revealed that a unique *CBR* gene copy was mutated in each case (Fig 4). We evaluated the effect of both mutations on carotenoid and sterol production at two points of the growth curve, and for comparative purposes, the wild-type and the *crtR* ^-^ mutant strains were included, which were cultured in parallel with the other two strains; the results were summarized in Table 2. The CBS-*cbr*.1^-^ strain carotenoid production was 3-fold higher than the wild-type strain at 72 h, while strain CBS-*cbr*.2^-^ did not show significant differences; in both mutant strains, the carotenoid composition was similar to that of the parental strain, and astaxanthin was the main carotenoid. The two *cbr*- mutants and the wild-type strain showed a similar sterol production and composition at both culture times, with ergosterol (approximately 98%) being the main sterol. These results indicate that even though one of the two *CBR* genes products could be involved in ergosterol biosynthesis, *crtR* complemented the absence of the CBR gene in the mutant strains, at least for ergosterol production. It would be very informative to obtain a *crtR* and *CBR*.1 double mutant strain; however, this might not be possible as in *S*. *cerevisiae* it has been shown that the double mutant of the cytochrome P450 reductase and cytochrome b5 encoding genes is not viable [48]. Considering this and to confirm if CrtR and CBR.1 mediate the oxidation of sterols, future *in vitro* enzymatic reaction experiments with CYP51 or CYP61 coupled with CrtR and CBR.1 could be performed.

**Table 2:** Carotenoids and sterols produced by the *X*. *dendrorhous* strains cultured in parallel after 36 and 72 h of cultivation.

	Metabolite production			
Strain	Total carotenoids (μg carotenoids/g dry yeast weight)		Total sterols (mg sterols/g dry yeast weight)	
	36 h	72 h	36 h	72 h
CBS 6938	57.5 ± 4.7	62.4 ± 3.5	7.8 ± 0.4	4.0 ± 0.6
CBSTr	46.4 ± 8.3	82.9 ± 4.4*	6.0 ± 1.0	4.0 ± 0.3
CBS-*cbr*.1^-^	61.0 ± 16.4	224.2 ± 4.5*	5.8 ± 0.3	4.2 ± 0.3
CBS-*cbr*.2^-^	54.2 ± 18.6	91.0 ± 21.3	5.5 ± 0.1	3.9 ± 0.4

* Statistically significant differences between the wild-type and mutant strains (Student’s t test, p<0.05 and α = 0.05).

### Cytochrome c reductase activity and gene expression in mutants and wild-type strains

The cytochrome c reductase activity assay is a useful approximation to evaluate cytochrome P450 reductase activity with NADPH as a reductant, as cytochrome c is an artificial substrate for CPR. Additionally, it has been shown that in some higher eukaryotes, CBR reduced cytochrome c with NADH only in the presence of CYB5 [49,50]; in others cases, it may do this directly [51,52]. Therefore, even though the cytochrome c reductase assay may not encompass the functionality of the complete CBR-CYB5 electron donor pathway, it is a useful approximation to determine cofactor preference in our mutants. In this way, indications of the contribution of each electron transfer via: NADPH or NADH for CPR or CBR-CYB5, respectively, can be obtained to determine if they are operative in *X*. *dendrorhous* microsomal extracts.

**Fig 5:**
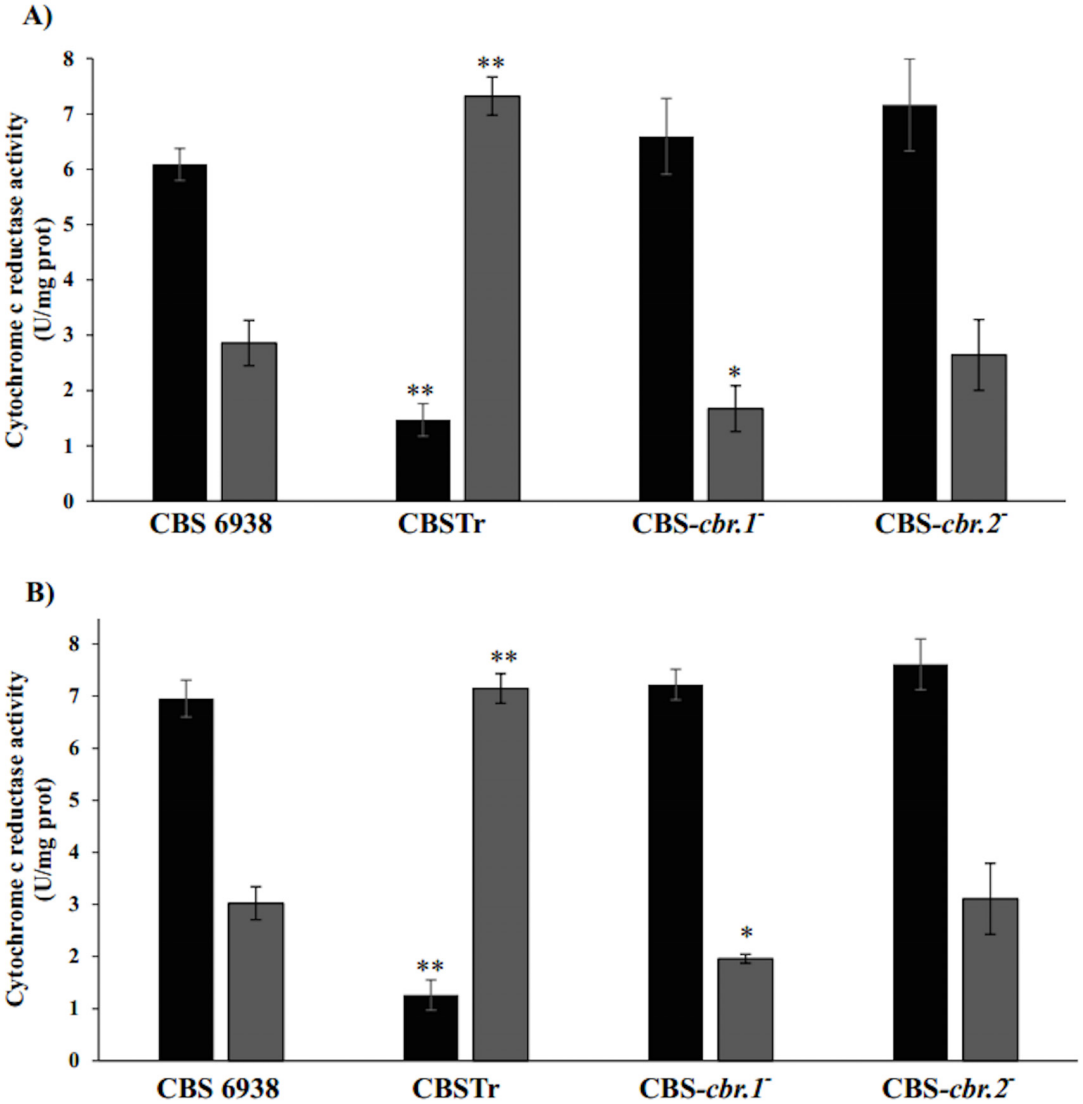
Cytochrome c reductase activity of the wild-type and mutant strains. Assays were performed with NADPH (black bars) or NADH (grey bars) as cofactor using microsomal fractions extracted after (A) 36 h and (B) 72 h of culture. Values are the mean ± standard deviation of two technical replicates from three independent cultures. Statistical significant differences between wild-type and mutant strain compared to the same assay are indicated (Student’s t test, *P<0.05, **P<0.01 with α = 0.05).

To evaluate the functionality of these enzymes in *X*. *dendrorhous*, cytochrome c reductase activity assays were performed with microsomal extracts from *crtR* ^-^ (CBSTr), CBS-*cbr*.1^-^, and CBS-*cbr*.2^-^ mutants and the wild-type strain using NADPH or NADH as cofactors. The microsomal fraction was obtained after 36 and 72 h of cultivation from the same three replica cultures used for metabolite production analyses in the four strains; the results were summarized in (Fig 5). In general, the microsomal fraction obtained from the wild-type strain at all cultivation times showed a higher cytochrome c reductase activity with NADPH than with NADH as a cofactor. However, the microsomal fractions from strain CBSTr showed an inverse pattern; they had higher cytochrome c reductase activity with NADH than with NADPH. Moreover, by comparing the activity results between these two strains, microsomes from CBSTr showed on average approximately 3-fold less activity with NADPH, whereas with NADH, the activity was almost 2-fold higher in relation to the wild-type strain. These are interesting results revealing that in order to reduce cytochrome c, microsomes from the wild-type strain have a preference for NADPH, while samples from CBSTr have a preference for NADH, which agrees with the absence of CPR. Similar to the wild-type strain, microsomes from the CBS-*cbr*.1^-^ and CBS-*cbr*.2^-^ mutant strains also showed higher cytochrome c reductase activity with NADPH than with NADH; however, microsomes from CBS-*cbr*.1^-^ showed lower NADH-dependent cytochrome c reductase activity in relation to the wild-type strain, while microsomes from CBS-*cbr*.2^-^ did not show significant differences (Student’s t test, p<0.05). As a whole, these results suggest that *crtR* in the CBS-*cbr*.1^-^ and CBS-*cbr*.2^-^ mutant strains contributes by sustaining the NADPH- dependent cytochrome c reductase activity, which could be sufficient to maintain the wild-type ergosterol production level. However, the reduced NADH-dependent cytochrome c reductase activity in CBS-*cbr*.1^-^ strongly suggests that CBR.1, and not CBR.2, is involved in the *X*. *dendrorhous* class II P450 systems, supporting the results from the bioinformatics analyses.

**Fig 6:**
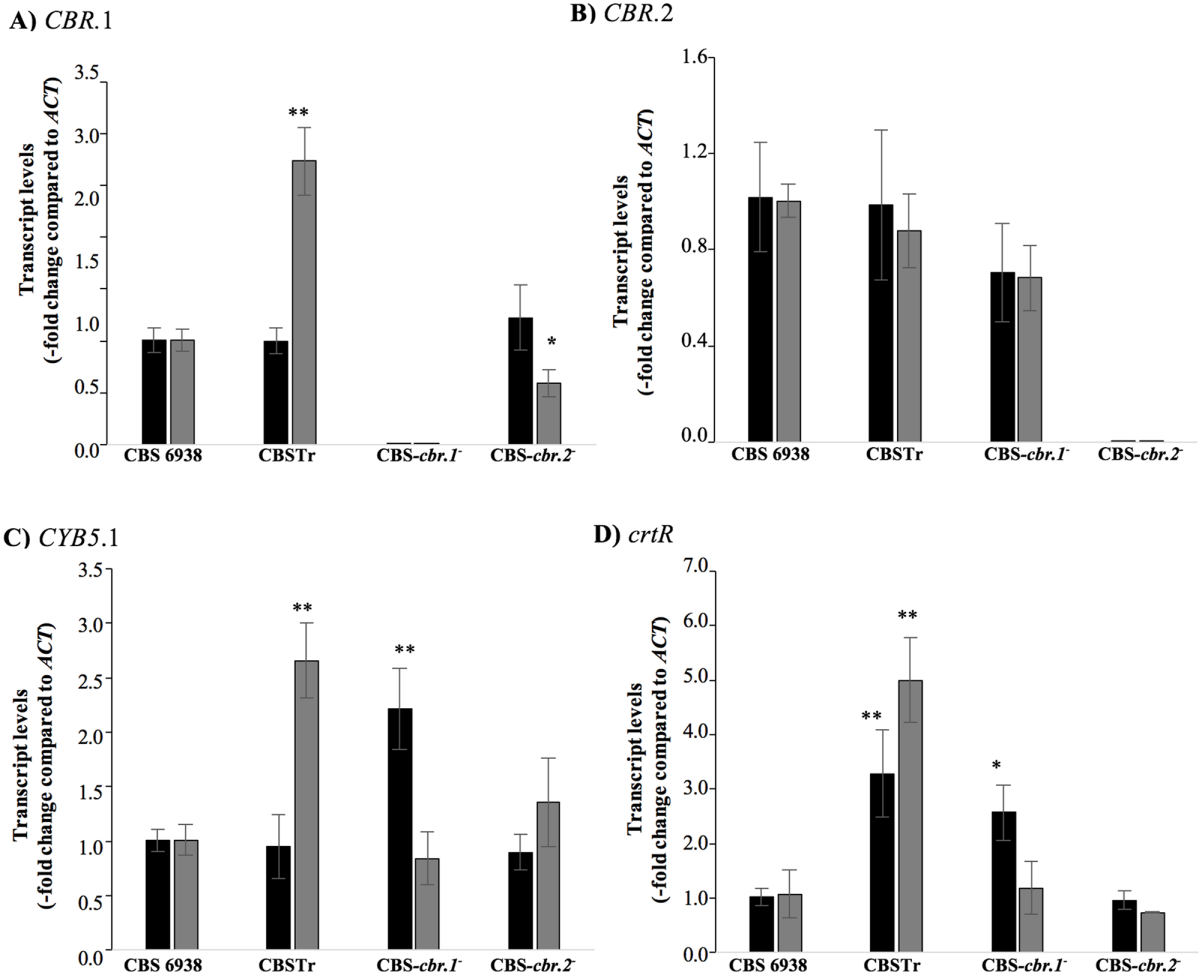
RT-qPCR for *CBR*.1. *CBR*.2, *CYB5* and *crtR* transcript levels in the wild-type and mutant strains. (A) *CBR*.1 (B) *CBR*.2, (C) *CYB5* and (D) *crtR* genes. The transcript level of each gene was normalized to actin mRNA. Black and grey bars represent the results using RNA extracted after 36 and 72 h of culture, respectively. Values are the mean ± standard deviation of two technical replicates from three independent cultures (Student’s t-test was performed to compare each mutant to the parental strain. * P <0.05 and ** P <0.01).

Considering the higher NADH- and the lower NADPH-dependent cytochrome c reductase activity in the *crtR* ^-^ mutant strain in relation to the wild-type strain, we evaluated if this could be a consequence of differential transcript levels of the studied genes among the analyzed strains in this work. Total RNA was extracted at the same time-points of the same cultures used in the previous studies, and *crtR*, *CBR*.1, *CBR*.2 and *CYB5* transcript levels were evaluated by RT-qPCR (Fig 6). As expected, the CBS-*cbr*.1^-^ and CBS-*cbr*.2^-^ strains did not show *CBR*.1 and *CBR*.2 transcripts. The transcript levels of the *CBR*.2 gene did not show significant differences between the analyzed strains, which was consistent with the previous results obtained in this work suggesting that the *CBR*.2 gene is not involved in the class II P450 systems. In the CBS-*cbr*.2^-^ strain, only *CBR*.1 showed a lower transcript level in relation to the wild-type strain after 72 h of culture. Interestingly, even though no differences were detected at the early stationary phase (36 h) of growth, the higher NADH-dependent cytochrome c reductase activity observed in microsomes obtained from CBSTr correlated with higher *CBR*.1 and *CYB5* transcript levels in relation to the wild-type strain after 72 h of culture. This observation could be explained as a compensatory mechanism for cytochrome P450 activity due to the *crtR* gene mutation in the CBSTr strain. This idea was also supported by the fact that there was a higher NADH-dependent cytochrome c reductase activity in the CBSTr strain, suggesting that in the absence of a functional CPR, the activity of an alternative P450 electron donor, likely CBR-CYB5 via, was enhanced in *X*. *dendrorhous*.

Finally, the transcript level of the *crtR* gene was determined. This gene was not completely deleted in the CBSTr strain, but it was interrupted with a module that confers resistance to hygromycin B [22]. Even though the gene product was not functional, it can be transcribed, allowing us to include it in these analyses. In the two culture time-points of growth analyzed, the *crtR* transcript levels in CBSTr were higher than in the wild-type strain. Considering that the sterol composition was altered in this strain, the reduced ergosterol content could enhance *crtR* gene expression as has been reported for other genes [18]. Interestingly, the transcript level of this gene was also higher than the wild-type strain in the CBS-*cbr*.1^-^ strain after 36 h of culture, suggesting that *crtR* could compensate for the absence of the *CBR*.1 gene in cytochrome P450 activity. Previously it was reported that the regulation of the expression of the cytochrome P450 reductase gene, which encodes the main electron donor in P450s systems, was particularly complex and involved several regulatory elements in the promoter region, differential promoter use and regulation at the post-transcriptional level [53,54]. As such, it was expected that the regulation of alternative redox partners such as CBR-CYB5 could also be subject to complex regulatory mechanisms. However, the mechanism by which the *crtR* ^-^ gene mutation affects the *CYB5* and *CBR*.1 transcript levels requires further investigation.

## Conclusions

In addition to being essential for astaxanthin biosynthesis, our results show that the *X*. *dendrorhous crtR* gene is also involved in ergosterol biosynthesis, as the ergosterol proportion was reduced in the *crtR* ^-^ mutant. The higher transcript level of the *CBR*.1 and *CYB5* genes and the increased NADH-dependent cytochrome c reductase activity in the *crtR* ^*-*^ mutant strain, as well as the reduced NADH-dependent cytochrome c reductase activity in the CBS-*cbr*.1^-^ mutant strain, all strongly suggest the involvement of CBR.1-*CYB5* as an alternative electron donor to P450 enzymes during sterol biosynthesis in *X*. *dendrorhous*.

## Supporting Information

S1 Table Primers designed and used in this work. Additional supporting information may be found in the online version of this article at the publisher’s website.
(PDF)
