# Supplementary material for: Molecular Characterization and Functional Analysis of Cytochrome b5 Reductase (CBR) Encoding Genes from the Carotenogenic Yeast Xanthophyllomyces dendrorhous
Source: PLoS One. 2015 Oct 14;10(10):e0140424. doi: 10.1371/journal.pone.0140424 (PMC4605618; doi:10.1371/journal.pone.0140424)
Supplement: S1 Table — Additional supporting information may be found in the online version of this article at the publisher’s website. (PDF) [file pone.0140424.s001.pdf]

Table S1. Primers designed and used in this work.

	Primer:	Sequence 5' a 3'	Target and/or special features
cDNA and gDNA amplification			
1	pre_gCBR1-Fw	GCG CAC AAT GAC TGA AGG CTT	*CBR.*1 gene
2	post_gCBR1-Rv	AAC CTA CAC AGA AGC TCT TGC	*CBR.*1 gene
3	gCBR1-HpaI-Fw	CTT CAT CTC TCG TTA ACA CAT CAA ACA AAA CCC ATG A	*CBR.*1 gene
4	gCBR1-HpaI-Rv	TTG TTT GAT GTG TTA ACG AGA GAT GAA GGG AGC AAG G	*CBR.*1 gene
5	CTR_gCBR1-Fw	GTA CCC GTT GAT CAA GCG AGT	*CBR.*1 gene
6	CTR_gCBR1-Rv	CAA GCT CGT CAA GTT CCT TCT	*CBR.*1 gene
7	pre2-gCBR1_Fw	TCC GCA CAT ATC GTC CAA GTT C	*CBR.*1 gene
8	post2_gCBR1-Rv	TGC TTC AAA CAG ACT CTA GAG G	*CBR.*1 gene
9	del_gCBR1-Fw	GTC GCT GTG ACA TGC TGA TGT	*CBR.*1 gene
10	del_gCBR1-Rv	TCT TAT TCC GCC CTT CCT ATC	*CBR.*1 gene
11	cytb5Red_del-Fw	GTG GTG AGT CCT TTG ATC TGT	*CBR.*2 gene
12	cytb5Red_del_HpaI-Fw	CAT CAC CCA TCG TTA ACA TTT GCC GCT AGA TGT TGA A	*CBR.*2 gene
13	cytb5Red_del_HpaI-Rv	GCG GCA AAT GTT AAC GAT GGG TGA TGT GAT GTG ATG	*CBR.*2 gene
14	cytb5Red_del-Rv	TGT ATC TTC CAG TCC GGT CTC	*CBR.*2 gene
15	CTR_cytb5Red-Fw	CTT GAC GCT CTC TAA GGT TGA	*CBR.*2 gene
16	CTR_cytb5Red-Rv	ACG CCA CAG ACA AAC ACC TTG	*CBR.*2 gene
17	pre_cytb5Red_del_Fw	GTT CCA AGT CCC CGG TAT CTA	*CBR.*2 gene
18	post_cytb5Red_del_Rv	AGA AAG ACA ACA GGA GGT AGG	*CBR.*2 gene
19	Zeo.F	GAC TTC GTG GAG GAC GAC TT	*Sh ble* gene
20	Zeo.R	CGC TGA TGA ACA GGG TCA C	*Sh ble* gene
21	H-out.F	CTC GAT GAG CTG ATG CTT TG	*hph* gene
22	H-out.R	TCC ATC ACA GTT TGC CAG TG	*hph* gene
**RT-qPCR analysis:**			
23	crtR_RT.F	TGTGTTTGGCCTGGGAAACAAGAC	*crtR* gene
24	crtR_RT.R	TGTCGTAACCGAGGTTCCTAACCA	*crtR* gene
25	cytb5_RT.F	CGTCCGATTCTGGAAAGATTACCC	*CYB5* gene
26	cytb5_RT.R	AAGCTGGTAAGGATGCGACTGA	*CYB5* gene
27	RT-CBR1.F	AAGCACATCGACGGACTCAA	*CBR.*1 gene
28	RT-CBR1.R	TTCCTCGGTCACATTAGCGT	*CBR.*1 gene
29	cytb5R_RT.F	CAAGGCTGGCTTCAAGGGTACATT	*CBR.*2 gene
30	cytb5R_RT.R	TCTTGCTTCGAAAGGAATGGGACG	*CBR.*2 gene
31	mactF-RT	CCGCCCTCGTGATTGATAAC	*ACT* gene
32	mactR-RT	TCACCAACGTAGGAGTCCTT	*ACT* gene
